# Effort-reward imbalance and self-rated health among Gambian healthcare professionals

**DOI:** 10.1186/s12913-016-1347-0

**Published:** 2016-04-11

**Authors:** Amadou Darboe, I-Feng Lin, Hsien-Wen Kuo

**Affiliations:** Ministry of Health and Social Welfare, Banjul, The Gambia; International Health Program, Institute of Public Health, National Yang-Ming University, Taipei, Taiwan; Institute of Public Health, National Yang-Ming University, Taipei, Taiwan; Institute of Environmental and Occupational Health Sciences, National Yang-Ming University, No.155, Sec.2, Li-Nong Street, Taipei, 112 Taiwan ROC; School of Public Health, National Defense Medical Center, Taipei, Taiwan

**Keywords:** ERI model, Healthcare workers, Sub-Saharan Africa, Gambia

## Abstract

**Background:**

The effort-reward imbalance (ERI) model of work stress has been widely applied in investigating association between psychosocial factors at work and health. This study examined associations between perceived psychosocial work stress as measured by the ERI model and self-rated health (SRH) among nurses and environmental health officers (EHOs) working in secondary public healthcare facilities in the Gambia.

**Method:**

A cross-sectional study on a random sample of 287 health care professionals (201 nurses and 86 EHOs). A 22-item ERI questionnaire was used to collect data on the psychosocial factors defined by the ERI model. SRH was assessed using a single item measure.

**Results:**

The distribution of subjective health was not statistically different between nurses and EHOs. However, our study uncovered significant associations between perceived psychosocial work stress and subjective health. Specifically, we found that a perceived high effort-reward imbalance (ER-ratio > 1) is a significant risk factor for poor SRH, in both occupational groups. However, over-commitment was not significantly associated with poor SRH in the two groups. When efforts and rewards were considered as separate variables in the analysis, rewards were inversely associated with poor SRH in both groups.

**Conclusion:**

Because of the high perceived Effort-Reward Imbalance among healthcare professionals at secondary public healthcare facilities, it is necessary to modify working conditions through improvement of psychosocial work environment, such as reasonable allocation of resources to increase pay, incentives or other forms of rewards from government. Interventions that could mitigate and prevent stress at work are worth considering in future healthcare policies.

**Electronic supplementary material:**

The online version of this article (doi:10.1186/s12913-016-1347-0) contains supplementary material, which is available to authorized users.

## Background

Health care workers are critical for the success of health systems and for the attainment of national and global health goals [1, 2]. The World Health Report 2006 defined health workers as people whose job it is to protect and improve the health of their communities [3]. Thus, to effectively respond to populations’ health needs, Health Care Professionals (HCPs) themselves must be in a perfect state of health devoid of morbid worries and anxieties [4]. This is important as health care is a very stressful profession and workers in healthcare are exposed to series of psychosocial stressors [4] including; shift work, high workload due to increasing demands, emotional distress due to interaction with patients and colleagues and low promotion prospects [5]. Constant exposure to these psychosocial hazards engender negative emotions leading to work related stress (psychosocial work stress) which has adverse consequence on subjective health and wellbeing of HCPs [5, 6]. In addition to influencing health and wellbeing of HCPs, psychosocial work stress may also influence patient care and treatment outcomes. For instance, evidence indicated that HCPs under stress perform poorly and are prone to making errors in clinical judgement [7].

In the Gambia, little attention has been given to the psychosocial work environment of HCPs. A survey by the Ministry of Health and Social Welfare (MOHSW) in collaboration with the West African Health Organization (WAHO), indicated that nurses working at secondary level of care have raised concerns regarding the improvement of their working environment [8]. Issues such as; delayed promotions, low salaries, lack of adequate resources, etc. appeared as challenges facing HCPs [8, 9]. Yet, no study has been done in the country that could inform policy about HCPs’ perceptions of their psychosocial work environment and how this is associated with their subjective health at work. Hence, questions such as: does the psychosocial work environment of HCPs constitute a perceived work stress and how this is associated with their subjective health at work cannot still be answered given the current state of knowledge in the Gambia. Therefore, the need for empirical data is eminent.

Thus, the present study has employed the Effort-reward Imbalance (ERI) model [10] to investigate the association between perceived psychosocial work stress and self-rated health (SRH) among HCPs in the Gambia. In its totality, the paper focused on two categories (cadres) of HCPs namely; healthcare nurses and Environmental Health Officers (EHOs) who serve at secondary level of healthcare in the Gambia. Basically, the Gambia’s healthcare delivery system is based on three tiers, namely; primary, secondary and tertiary level of healthcare. Secondary healthcare facilities generally comprise of health centers and clinics that provide primary health care services (PHCS) to communities [9]. Nurses form the highest proportion of healthcare professionals at this level of care followed by EHOs. Considering the critical role of HCPs, studying their SRH is imperative from both the development and health policy perspectives. A single item measure of self-rated health has been found to predict mortality and functionality even after accounting for other covariates [11]. Thus, this subjective measurement of health also helps policy makers to identify at risk individuals in the population for timely interventions [11].

The ERI model is a theoretical model widely used in measuring associations between perceived psychosocial work stress and health through identifying the mismatch between job demands (efforts) and benefits (rewards) in a work setting [12]. The model has defined the psychosocial work environment into three main components namely; efforts and rewards (the situational/structural factors) and over-commitment (personal factors). According to the model, efforts at work represent the demands and obligations that are imposed on an employee at work while occupational rewards represent job resources and benefits provided to the employee including money, esteem, and career opportunities [13]. The model hypothesized that, efforts and rewards may each predict poor health and wellbeing, however the imbalance between high effort and low reward (ER-ratio > 1) have stronger predictive effect on poor health and wellbeing over and above the effects of each single component [12]. This assumption (the ERI hypothesis) is the key theoretical construct of the ERI model. Over commitment on the other hand, is a personal trait that defines individual coping pattern with high efforts and low rewards condition at work [10]. It is stated that individuals characterized by high work related over commitment tend to have intense need for control, esteem and approval at work [14]. As a result, such people underestimate the demands at work and overestimate their own potentials and in the long run they are likely to experience reward frustration and exhaustion [15]. According to the ERI model, individuals characterized with high level of over commitment will have higher risk of poor health compared to their low overcommitted counterparts (the overcommitment hypothesis) [12]. However, this risk is expected to increase if ER-ratio and overcommitment components act in concert ( the interaction hypothesis) [12]. These three components (efforts, rewards and overcommitment) also represent the psychometric scales of the model [14] and were each studied separately in this paper to determine their association with self-rated health in the study population.

Previous studies using ERI model focused on work stress and health status among healthcare workers, emphasizing nurses and physicians [5, 6, 16]. Studies on effort-reward imbalance (ERI) and self-rated health (SRH) are scarce in the literature [5, 15, 17, 18], and the few that exist were conducted in high income countries. We came across only two studies in Sub-Saharan Africa that applied the ERI model [19, 20] and these studies differ in their methodologies. For example, Peltzer et al. [19] could not use the full scales of the ERI model in assessing job stress, job satisfaction or stress-related illnesses among South African educators. Despites the methodological differences, both studies have provided support to the assumptions of the model. Peltzer et al. [19] have found an inverse association between occupational reward (career advancement) and hypertension among South African educators. Furthermore, Ojedokun, [20], has found high effort-reward imbalance (ER-ratio > 1) to be a predictor of attitude towards unethical work behavior among police officers in Nigeria.

Hence, this paper has applied the full scale of the ERI model to obtain information regarding perceived psychosocial work stress among healthcare professionals in the Gambia. We were compelled to rely on this theoretical model due to its ability in considering contextual, economic and individual level factors related to employees’ health and wellbeing [21]. The present investigation has the following aims;

First, it attempts to provide a baseline information on the association between perceived psychosocial work stress as measured by ERI model and SRH (subjective health) of health care nurses and EHOs working in secondary public health care facilities in the Gambia. Second, to contribute to the present state of knowledge on ERI and self-rated health especially, from the context of a developing country like the Gambia where the issue remains under-researched.

## Methods

### Study design and sample

In this cross-sectional survey, we recruited trained healthcare nurses and environmental health officers who were working in secondary public healthcare facilities in both rural and urban areas of the Gambia. The Gambia has 6 political administrative and 7 health administrative regions of which, two are considered urban areas and the rest formed the rural areas. The seven health administrative regions include; i) Regional health management team (RHMT) Western I, ii) RHMT Western II, iii) RHMT Lower River Region, iv) RHMT Central River Region, v) RHMT North Bank East, vi) RHMT North Bank West, and vii) RHMT Upper River Region. The first two health regions on the list are characterized under urban health regions and the remaining 5 under rural health regions. Each RHMT is responsible for monitoring and supervising as well as budgeting basic resources for those health facilities within its jurisdiction [9]. According to the annual health service statistics report 2012, in total there were 41 minor & 6 major public health centers in the country [22] that provide services at secondary level. Further, there were 860 trained Nurses (including Registered Nurses, Enrolled Nurses and Community Health Nurses) and 117 EHOs working in government health centers and hospitals. Nurses represent approximately 75 % of the total healthcare workforce in the country [22] and are responsible for all clinical judgements at secondary level of healthcare. EHOs are responsible for preventive services, including but not limited to; provision of immunization services, disease surveillance, birth registrations and environmental inspections.

The sample size for this study was calculated using the online sample size calculator of Creative Research Systems. We selected a 95 % confidence level, a margin of error express as decimal was ±0.0478, response distribution was set to 50 % and populations sizes of 997, and eventually we arrived at an estimated sample size of 296. To select this sample from the population, a multi-stage random sampling technique was used. In this process, we first listed the facilities as clustered in either rural or urban settings. Then, by means of simple random sampling, we selected two health regions in rural areas (RHMT Upper River Region and RHMT North Bank West) and one in the urban areas (RHMT Western Region 1). Afterwards, we located all secondary public health facilities in all these health regions. We finally arrived at our sample base by contacting all trained Nurses and EHOs working in these health facilities at the time of the data collection. Initially, to ensure better response rate, we distributed 300 self-administered anonymous questionnaires to the healthcare professionals and with the assistance of the officers-in-charge (heads) of the facilities we were able to follow them. Participation in this study was entirely voluntary. In total, we visited 21 (15 rural and 6 urban) public secondary healthcare facilities between July and September 2014, and received affirmative responses from 290 professionals. Three of the questionnaires were dropped due to large amount of missing values (>50 %) hence, the remaining 287 questionnaires were from 201 nurses (106 females, 92 males and 3 with missing data for gender) and 86 environmental health officers (28 females and 58 males). The sample had a mean age of 32.4, and the total response rate was 97 %. Each self-administered questionnaire was issued with an information sheet and a consent form stipulating the purpose of the study and seeking their consent for participation. Copies of written consent forms were returned by all participants. Confidentiality in this study was ensured through the use of anonymous and self-administered questionnaires. Ethic approval was obtained from the Joint Gambia Government/Medical Research Council (MRC) Ethics Committee.

### Measurement

#### Dependent variable

In this study, the dependent variable was poor self-rated health and was assessed using a single-item measure with five response levels [11, 23]. More specifically, respondents were asked: “In general, how would you rate your health today”, with response options being “very good (1), good (2), moderate (3), bad (4) and very bad (5)” (cf. Subramanian et al. [23]) [24](Additional file 1). We analyzed SRH as a dichotomous variable with “moderate/fair, bad or very bad” coded as 1 and regarded as ill-health; “good or very good” was coded as 0 and regarded as healthy [11, 23, 24]. This approach (dichotomizing the scores) is in conformity with previous studies reporting SRH and was used here to enhance easier understanding regarding the distribution of subjective health in the study population.

#### Independent variables

##### The effort-reward imbalance model

We used the 22-item long version of ERI questionnaire (ERI-L version 22.11.2012) [25] (Additional file 1) to measure HCPs’ perceptions of their psychosocial work environment. The questionnaire employed was without alteration. In this questionnaire, an effort scale consisting of 6 items represents the demand of different aspects in the work environment (e.g., work pressure, time pressure, responsibility, working overtime, physical load and increasing demands). The other two scales, reward and overcommitment, consist of 10 items and 6 items, respectively. Reward has 3 sub-scales: promotion (4 items), esteem (4 items) and security (2 items). In our study, the internal consistencies of all the ERI scales were satisfactory: “0.86” for effort, “0.76” for reward and “0.70” for over-commitment. Effort, reward and over-commitment were all measured on a four-point response scale, ranging from 1 (strongly disagree) to 4 (strongly agree). For analysis purpose, the sum scores for effort and reward were each analyzed as continuous predictors in order to study their association with subjective health. Furthermore, scores for over-commitment were divided into tertiles, and exposure to high levels of overcommitment was defined as the upper tertile of the distribution among the total study population [15]. Higher mean scores of effort and over-commitment as well as lower mean scores of rewards were considered to indicate perceived psychosocial stress at work.

The ERI ratio for each study participant was computed using the sum scores for efforts as the numerator and the sum scores for rewards as the denominator multiplied by a correction factor of 0.6 [Effort/(Reward*0.6)]. This correction factor was used to adjust for unequal number of items in both scales [14, 26]. A ratio over one (ER ratio > 1) indicates an exposure to high Effort-reward Imbalance at work which also constitutes a perceived psychosocial work stress [10].

##### Covariates

To ascertain the association between perceived psychosocial work stress and subjective health, we adjusted for a range of potential confounders. These included age, gender, work site (e.g., urban or rural), smoking habits, education, occupational group, physical activity, marital status, years of service (seniority), level of dependency, number of stressful events experienced in the past month and chronic conditions (e.g., diabetes, asthma and hypertension).

#### Statistical analysis

We applied a variety of statistical measurements to paint a comprehensive picture of the association between effort-reward imbalances and self-rated health. It is important we mention that, though region (work site) may have an effect on the associations between perceived psychosocial work stress and subjective health due to single stage clustering (rural and urban), however we adjusted for this effect by controlling for region and several other demographic covariates in all relevant analysis.

Cronbach alpha (α) was used to assess the internal consistency of the ERI scales We used analysis of missing values in SPSS version 20 to assess the mechanism of missing data in our data-set. A non-significant Little’s MCAR test *χ2* = 72.45, p-value = 0.194 indicated that our data were missing completely at random (MCAR) hence, we used pairwise deletion technique (available-case analysis) to handle missing data in the analysis. If data are MCAR then missing data are ignorable and the use of pairwise deletion is expected to provide unbiased parameter estimates in the analysis [27].

All categorical covariates (e.g. gender, marital status, level of education, region etc.) were sent to Pearson’s χ^2^ test to examine their association with profession. Independent t-test was used to measure mean differences for continuous covariates (age and years of service). Furthermore, we used analysis of covariance (ANCOVA) adjusting for age and region (work site), in order to examine mean differences in ERI scales between the occupations. The rationale for adjusting for age in ANCOVA was because several studies have found low efforts to be associated with increasing age [18] and for region was to minimize any effect that may result due to clustering. In addition, we used Pearson Correlation analysis to measure associations between ERI scales and SRH.

In order to test the hypothesis of the ERI model with respect to subjective health, and to provide results comparable to previous publications [14], two main formulations of the model were studied using multiple logistic regression models adjusted for personal, occupational and health related covariates. In the first model, the ER-ratio and overcommitment, were simultaneously entered into the model as dichotomous (binary) variables as suggested by Siegrist et al. [14, 16]. In the second model, the sum scores for efforts, rewards, overcommitment and ER-ratio were simultaneously entered as continuous variables. Evidence from previous studies indicated that compared to binary measure, continuous measures of the ER-ratio provide more information and stronger statistical effects [14]. Due to available-case analysis approach, the results we presented here were based upon cases with complete data as incomplete cases were deleted in an analysis-by-analysis basis. Thus, only individuals with complete data on SRH (97 % of participants) were included in the multiple logistic regression model. All analyses were run on SPSS (version 20) and Stata (version 12.1).

## Results

We present results of Chi-square analysis and independent t-test in Tables 1 and 2. Table 1 compares the personal and health related characteristics between the two occupational groups. Of the 287 surveyed participants, Nurses were relatively older with a mean age of 34.7; additionally, a good proportion of Nurses were above 35 years of age whereas a considerable proportion of EHOs were under 30 years of age (all *p* < 0.001). The proportion of females and married professionals were higher among Nurses; 53.5 % and 74 % respectively (*p* < 0.001). Approximately 76 % of Nurses and 59 % of EHOs reported to be supporting two or more people beside their own families. About 14 % of the Nurses and 20 % of the EHOs were categorized as being of poor self-rated health, but the difference was not statistically significant across the two groups. No statistical significant association was observed between region and profession.

**Table 1:** Personal and health related characteristics by occupational category

Variables	EHOs (*N* = 86)		Nurses (*N* = 201)		*P*
	n	%	n	%	
Region					.767^a^
Urban	42	48.8	102	50.7	
Rural	44	51.2	99	49.3	
Gender					0.001^a^
Female	28	32.6	106	53.5	
Male	58	67.4	92	46.5	
Age group					<0.001^a^
< 26	33	39.3	37	19.3	
26-30	39	46.4	42	21.9	
31-35	8	9.5	31	16.1	
> 35	4	4.8	82	42.7	
Age (mean ± SD)	27.29 ± 3.87		34.68 ± 9.25		<0.001^b^
Marital Status					<0.001^a^
Yes	36	41.9	149	74.1	
No	50	58.1	52	25.9	
Dependency					0.004^a^
< 2 people	35	40.7	48	24.0	
≥ 2 people	51	59.3	152	76.0	
Stressful personal event					.682^a^
Yes	47	56.0	116	58.6	
No	37	44.0	82	41.4	
Smoking status					.073^a^
Yes	1	1.2	12	6.0	
No	85	98.8	189	94.0	
Exercise					.395^a^
Yes	49	57.0	103	51.5	
No	37	43.0	97	48.5	
Chronic disease					.167^a^
Yes	4	4.7	19	9.5	
No	82	95.3	181	90.5	
Self-rated health					.208^a^
Poor	17	19.8	27	13.8	
Good	69	80.2	168	86.2	

^a^Pearson’s χ^2^ test, ^b^Independent t-test

**Table 2:** Comparison of occupational characteristics

	EHOs (*N* = 86)		Nurses (*N* = 201)		*P*
	Mean	SD	Mean	SD	
Length of service (in yrs.)	3.09	2.24	10.04	9.07	<0.001^b^
	n	%	n	%	
Educational level					<0.001^a^
Certificate	.	.	175	87.5	
Diploma	77	89.5	19	9.5	
Degree	9	10.5	6	3.0	
Part-time job					.172^a^
Yes	6	7.0	25	12.4	
No	80	93.0	176	87.6	
Hours at work					.908^a^
≤ 40	38	44.7	90	45.5	
> 40	47	55.3	108	54.5	
Shift work					<0.001^a^
Yes	21	24.7	127	63.8	
No	64	75.3	72	36.2	
ER-ratio					
< 1	25	29.4	26	13.2	0.001^a^
> 1	60	70.6	171	86.8	
Overcommitment					0.001
Low (lower tertiles)	66	76.7	110	55.6	
High (upper tertile)	20	23.3	88	44.4	

^a^Pearson’s χ^2^ test, ^b^Independent t-test

Table 2 compares the occupational characteristics between the two professions. The two professions differ significantly in their level of education *(p* < 0.001), with approximately 88 % of Nurses having only certificates, while about 90 % of EHOs had diplomas. The mean length of service for Nurses was 6.95 higher than that of EHOs (p < 0.001). Shift work was at 63.8 % among Nurses and at 24.5 % among EHOs. With regard to psychosocial variables, we observed a high prevalence of perceived effort-reward imbalance (ER-ratio > 1) in the two groups, however the percentages was higher among Nurses (86.8 %) than EHOs (70.6 %) (*p* = 0.001). We also observed that the level of work related overcommitment was significantly higher among Nurses (44.4 %) than among EHOs (23.3 %) (*p* = 0.001),

Table 3 displays the means and standard deviations of ERI scales**.** After adjusting for age and region in the analysis, we observed a significant difference in the means of all the scales across the two occupational groups. Comparatively, Nurses reported higher mean scores of psychosocial work stress.

**Table 3:** Comparison on psychosocial factors in the workplace

Psychosocial Factor	EHOs(*n* = 86)		Nurses (*n* = 201)		P*	Total	
	Mean	S.D	Mean	S.D		Mean	SD
Effort	17.99	3.18	18.17	3.25	0.02	17.80	3.32
Reward	24.39	3.94	22.80	4.59	0.02	23.31	4.41
Overcommitment	15.70	2.73	16.93	2.91	0.001	16.57	2.91
Effort-reward ratio	1.20	0.35	1.41	0.46	0.003	1.35	0.46

*Difference between Environmental Health Officers and Nurses, ANCOVA adjusted for age and region

Table 4 shows Pearson’s correlation coefficients for the SRH and ERI scales. Among EHOs, SRH was negatively correlated with ER-ratio and effort but positively correlated with occupational rewards (all statistically significant *p* < 0.05). Among Nurses, a significant negative correlation was observed between SRH and ER-ratio (*p* < 0.05). In both occupational groups, ER-ratio was negatively correlated with rewards but positively correlated with efforts and overcommitment (all *p* < 0.01). A negative significant correlation was observed between rewards and overcommitment in the nursing group only (*p* < 0.05).

**Table 4:** Correlation analysis of self-rated health and ERI scales by occupational category

Variables	Groups	Variables by number				
		1	2	3	4	5
1. Self-rated Health	EHOs	1				
	Nurses					
2. Efforts	EHOs	-.24*	1			
	Nurses	-.10				
3. Rewards	EHOs	0.33**	-.29**	1		
	Nurses	.11	-.21**			
4. Overcommitment	EHOs	.03	.41**	-.10	1	
	Nurses	-.11	.45**	-.16*		
5. ER-ratio	EHO	-0.41**	0.82**	-0.76**	0.32**	1
	Nurses	-0.20*	0.67**	-0.80**	0.34**	

Pearson’s correlation coefficient sig. * *p*-value < 0.05 **p-value < 0.01

In Table 5, we verified two hypothesis of the theoretical model using adjusted multiple logistic regression analysis namely; the ERI hypothesis and the overcommitment hypothesis. By dichotomizing the scores, it appeared that healthcare professionals who reported high perceived efforts in combination with low rewards at work (ER-ratio >1) were nearly 4 times more likely to report a poor SRH after holding other covariates constant. Likewise, the continuous form of the ratio (ER continuous ratio) also indicated that by adjusting for other covariates in the model, per point increase in ER-ratio was associated with approximately a 3 folds increase in odds (chances) of reporting a poor SRH among HCPs. While these two odds ratios (ER-ratio binary and continuous ratio) differ in absolute terms and in terms of interpretation, yet, they both point to the same conclusion that high ER-ratio is a significant predictor of poor SRH among HCPs. However, neither the binary no the continuous formulations of overcommitment were significantly associated with poor SRH. By considering efforts and rewards as two separate variables in the model, rewards were inversely associated with poor SRH in both professions even after accounting for other covariates in the model. However, no statistical association can be found between efforts at work and poor SRH in the two groups.

**Table 5:** Effort-reward imbalance and overcommitment as risk factors for self-rated health: a multiple logistic regression analysis adjusted for personal factors (age, gender, marital status, no. of stressful events and level of dependency), occupational factors (education, work location, shift, hours of work, length of service), previous illnesses (diabetes, hypertension, asthma, etc.) and behavioral (smoking status and physical activity)

Parameter	Degree	Total Sample (*N* = 281)	EHOs (*N* = 86)	Nurses (*N* = 195)
		OR(95 % CI)	OR(95 % CI)	OR(95 % CI)
Binary scales				
ER-ratio	≤1	1.00	1.00	1.00
	>1	3.67 (1.57, 8.59)**	4.79 (1.23, 18.68)*	3.29 (1.11, 9.78)*
Overcommitment	Low	1.00	1.00	1.00
	High (upper tertile)	1.43(0.63, 3.22)	1.77(0.34, 9.12)	1.21(0.39, 3.72)
Continuous scales				
ER-ratio	(Continuous)	2.81 (1.34, 5.90)**	10.56 (1.74, 63.91)**	2.50 (1.03, 6.10)*
Overcommitment	(Continuous)	1.05 (0.90, 1.23)	0.96 (0.75, 1.23)	1.09 (0.89, 1.34)
Efforts	(Continuous)	1.04 (0.90, 1.20)	1.09 (0.86, 1.39)	1.01 (0.83, 1.23)
Rewards	(Continuous)	0.87 (0.79, 0.95)**	0.80 (0.66, 0.97)*	0.87 (0.77, 0.98)*

*p-value <0.05, **p-value <0.01

## Discussion

In the Gambia, the need for better psychosocial conditions at work have been a concern among HCPs in secondary public healthcare facilities for quite a while [8, 9]. Yet, policy makers are not informed about the magnitude of the problem among HCPs. Hence, our study aimed at bridging this knowledge gap by providing a baseline information on the association between perceived psychosocial work stress, as measured by the ERI model [10], and poor SRH in a sample of Nurses and EHOs working in secondary public health care facilities in the Gambia. Based on this model of work stress, we hypothesized that an imbalance between high efforts (job demands and obligations) and low rewards (job resources and benefits) (i.e. ER-ratio > 1) would predict poor SRH. We also posited that being highly over-committed (desire for esteem, control and approval) could predict poor SRH.

In our study, different components of the work environment that may constitute a perceived psychosocial stress were measured and their associations with SRH have been examined. We found overall; 15 % prevalence of poor SRH, and 87 % prevalence of perceived effort-reward imbalance among the HCPs. Relatively, nurses reported higher perceived efforts, gain fewer rewards and express a higher degree of overcommitment at work compared to EHOs. We also found that in both occupational groups, SRH show a significant negative correlation with ER-ratio. Results from logistic regression analysis suggested that both the binary and continuous formulations of the ER-ratio are significant predictors of poor SRH among HCPs (nurses and EHOs). Furthermore, occupational rewards were inversely associated with poor SRH among HCPs. However, over-commitment and efforts were not significantly associated with poor SRH among HCPs, a fact counter to our predictions.

This latter finding provides an evidence contrary to the assumption of the ERI model, that high level of overcommitment (need for esteem and approval) at work predicts poor health [12]. This may be explained on the basis of previous assumptions; that the desire to be esteemed and approved in a workplace are high order social needs that can only matter to employees when basic needs such as salaries, career opportunities, security etc. are satisfied [6]. Thus, considering the situation of the Gambia, where salaries of HCPs does not keep pace with inflation and are considered low [8] and where demands for timely promotion, increase wages, and better housing conditions were of concerns among HCPs [8, 9], hence, we could expect the effect of ER-ratio on subjective health to be stronger and more prominent than that of overcommitment.

Our findings indicate that HCPs in our study population perceived high efforts and moderate rewards which translates to the mismatch between efforts they expensed and rewards they gained at work. Theoretically, this findings provide evidence suggesting the presence of psychosocial stress among HCPs in secondary public health facilities in the Gambia. According to the ERI model, perceived failed reciprocity (an imbalance) between efforts and rewards in a work setting produce emotional distress with special propensity to autonomic arousal and associated strain reactions among workers (cf. Siegrist, 1996) [10]. The higher observed perceived psychosocial stress at work in Nurses compared to the EHOs is perhaps due to the work characteristics of the Nurses. Nurses tend to work under the pressure of trying to deal with different categories of patients throughout the year.

Our findings show inverse (negative) association between perceived occupational rewards and poor subjective health among secondary public healthcare professionals in the Gambia. This implies that as perceived occupational rewards increase, the odds (chances) of reporting poor subjective health among HCPs decreases. This result substantiates those reported from the Republic of South Africa [19] the Netherlands [6] and France [15]. It appears that analyzing occupational rewards in this way (as a continuous predictor) also provides meaningful information for identifying possible dose response association [14] between rewards and health.

Likewise, our finding that HIGH effort-reward ratios (ER-ratios binary and continuous) predict poor SRH has confirmed those from previous studies reporting SRH [5, 15, 17]. Putting them together, our findings highlight the significance of reciprocity in a healthcare setting. A lack of adequate compensation at work engenders failed reciprocity between perceived efforts and rewards, in the long-run arousing negative emotional responses [12] that in turn lead to stress-related mental and physical impairments [28]. Thus, it is the imbalance (failed reciprocity) that matters most [12].

Collectively, these findings are very important in the context of Sub-Saharan Africa where the retention of HCPs remains a major challenge for health systems; and where the protection of health and wellbeing of health workers remains a neglected part of healthcare priorities [29]. In this sub region, the healthcare systems are experiencing increasing shortage in skilled staffs, brain drain, rising levels of dissatisfaction and diminishing motivation [3] as well as increasing burden of infectious diseases [2]. These situations are the consequences of inadequate compensation and incentives (financial and otherwise), unsafe working environment, and insufficient or no career development opportunities [2].

As a Sub-Saharan African country, the Gambia’s public health sector is no exception to the aforementioned challenges. In fact, the Ministry of Health has to an extent, implemented financial incentives, hoping that these incentives would solve its human resource bottleneck. However, a study conducted by the Public Health Research & Development Centre in the Gambia reported that only 36 % of the 122 healthcare workers expressed their satisfaction with the incentives they are receiving, instead, they raised concerns regarding the improvement of their working environment [8]. This indicates that financial incentive is merely one piece of a broad occupational reward continuum.

Findings of this study have suggested that occupational rewards in terms of money (adequate salaries), career opportunities (job security, promotion prospects), and esteem (respect and support at work) are important not just for retention purpose but for the maintenance of a healthy workforce. Furthermore, the prevalence of high perceived mismatch between high efforts and low rewards in the study population suggests the need for interventions that could reduce and control work-related stress in healthcare. Amidst the high burden of infectious diseases, increasing demands for healthcare and the traumatizing public health events (e.g. disease outbreak such as Ebola etc.) witnessed by HCPs in Sub-Saharan Africa, the need for such interventions are high. For instance, supportive supervisions of HCPs has been linked to improved job satisfaction and low turnover intention in three African countries- Malawi, Tanzania, and Mozambique [7].

### Strengths & limitations

One of the strengths of our study lies in our applying the full test of the ERI model. Through doing so, we obtained results that are consistent and comparable with those from previous studies. In addition, our study is the first in the Gambia to examine the association between perceived psychosocial work stress and the subjective health of healthcare workers. It is also among the first studies in Sub-Saharan Africa to apply the ERI model of work stress in a healthcare. Because our data on the perceived psychosocial work were based upon subjective appraisals by the study subjects, our dataset represents an accurate and fair evaluation of the perceived work environment. In addition, our use of a single item measure of general health has made response to the questionnaires less cumbersome, resulting in a better-than-average response rate. Furthermore, we controlled for a number of personal, occupational and health related confounding variables, thereby adding much validity to the associations reported. Finally, because our study employed a random sample of public healthcare facilities from both rural and urban areas, our findings can be generalized to all secondary public healthcare facilities in the country.

Despite the many strengths of our study, it is not without limitations. Our study focused on nurses and environmental health officers in secondary public healthcare facilities, thereby limiting the generalizability of our findings to hospitals and private health care facilities. Also, due to the cross-sectional nature of the study, we cannot claim the associations we observed between perceived psychosocial work stress and subjective health to be causal. Our reliance on self-reported data for measuring general health and psychosocial stress might have led respondents to report underestimations or overestimations due to the social desirability bias or negative affectivity [15]. However, we partly addressed this issue by adjusting for personal events and overcommitment scale, which incorporates information pertinent to personality [15]. Persons with negative affectivity are likely to overestimate their exposure to psychosocial work stress and underestimate their health status [30]. Adjusting for stressful events (e.g. marital separation, conflict with the management, spouse etc.) in the logistic regression analysis sought to address this bias. On the contrary persons characterized with social desirability trait (need for social approval and acceptance) are likely to provide positive responses regardless of their true feelings [31] about their health and work environment. Hence, adjusting for overcommitment scale in the logistic regression analysis was an attempt to reduce this bias. Furthermore, information bias might have been present as a result of the “healthy worker effect”: those subjects who were on sick leave could not have been captured in our sample. Nevertheless, this has likely biased our results towards the null, as the bias should have led to an underestimation of the true association between psychosocial work stress and subjective health.

## Conclusion

We drew three main conclusions based on our findings. First, the main theoretical assumption of the ERI model of work stress holds. We affirmed that high effort-reward imbalance (ER-ratio > 1) at work is associated with poor subjective health among HCPs. Hence, our study has added to the growing body of empirical knowledge on ERI and SRH. Second, our data confirmed a high prevalence of psychosocial work stress among HCP in secondary public healthcare facilities of the Gambia. Third, the psychosocial work stress experienced in secondary public healthcare facilities is associated with poor subjective health among HCPs. Due to the high degree of mismatches between perceived effort expended and the rewards gained, governments must ensure better compensation schemes for healthcare workers, schemes that take into account, the broader psychosocial needs of workers. Implementation of stress management plans in healthcare could be useful for the welfare of HCPs.

We suggest future inquiries to consider including in the sample; all physicians and nurses in public and private health centres and hospitals to ensure more diversity and generalizability to other levels of healthcare and sectors. At design level, these inquiries may consider mix methods (quantitative and qualitative methods) to ensure in-depth discussion on the matter. Using statistical modellings substantiated with qualitative data can indeed generate more information for policy consideration.

## Additional file

Additional file 1: Questionnaire for the Investigation of Effort-reward Imbalance and Self-rated Health among Gambian Healthcare Professionals. (DOC 616 kb)

## Abbreviations

ANCOVA: analysis of covariance
CARD: center for advocacy and research development
ER: effort-reward
ERI: effort-reward imbalance
ERI-L: effort-reward imbalance questionnaire long version
HCP: health care professionals
MCAR: missing completely at random
MOHSW: Ministry of Health and Social Welfare
MRC: medical research council
PHCS: primary health care services
RHMT: Regional Health Management Teams
SRH: self-rated health
WAHO: West African Health Organization
