# Supplementary material for: Effort-reward imbalance and self-rated health among Gambian healthcare professionals
Source: BMC Health Serv Res. 2016 Apr 11;16:125. doi: 10.1186/s12913-016-1347-0 (PMC4828755; doi:10.1186/s12913-016-1347-0)
Supplement: Additional file 1: — Questionnaire for the Investigation of Effort-reward Imbalance and Self-rated Health among Gambian Healthcare Professionals. (DOC 616 kb) [file 12913_2016_1347_MOESM1_ESM.doc]

Questionnaire for the Investigation of Effort-reward Imbalance and Self-rated Health among Gambian Healthcare Professionals

**Personal & Health Related Questions**

We kindly ask you to answer the following questions and statements. By doing so you contribute to a better understanding of the situation of the healthcare workforce in the Gambian. We thank you in advance!

| 1. What is the year of your birth? | 19_____ year |
| --- | --- |
| 2. What is your gender? | *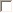* Male 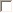 Female |
| 3. What is your level of education? | - Masters, 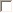Bachelor 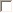Certificate 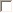 Diploma |
| 4. Which occupational group do you belong? | - Registered Nurse 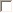 Enrolled Nurse - Community Health Nurse 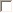Public Health Officer |
| 5. What is your marital status? | - Married 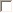Single 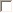Other |
| 6. Are currently an officer in Charge (OIC)? | 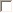 Yes 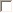 No |
| 7. Which region of the country do you work? | 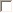Rural 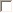Urban 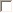Semi-urban |
| 8. How many years have you worked in your current employment? | ______ years |
| 9. Apart from your main employment, do you have any other jobs (part-time job)? | *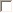*yes 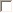No |
| 10. In total, how many hours in a week do you spend working for pay? | 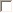Less than 40 hours 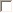Exactly 40 hours 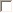 More than 40 hours |
| 11. Are you scheduled on shift work? | 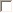 yes, but without nightshift 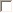 yes, with night shift 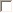 No |
| 12. Apart from your immediate family do you have other people to take care of? | - Yes 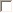 No |
| 13. If yes how many are they? | - One person, 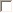 two people 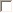 more than two people |
| 14. Do you currently smoke tobacco? | - Yes 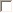No 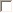 Quit smoker |
| 15. How many packs do you smoke daily? | - Less than one pack, 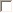 one pack 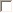 more than one pack   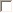 Don’t know |
| 16. Do you experience any stressful event for the past months such as conflict, loss of a close relative, etc. | - Yes 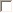 No |
| 17. How many stressful events have experienced for the past month | - Only one 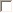 Two, 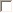More than two,   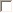 Don’t remember |
| 18. Have you ever been diagnosed by a doctor of any chronic diseases such as: diabetes, cardiovascular diseases, asthma, etc.? | - Yes 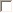 No |
| 19. How many chronic diseases were you diagnosed of? | - One 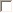 two 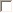 more than 2 |
| 20. Do you do regular exercise in your leisure-times? | - Yes 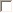 No |
| 21. In a week during your leisure-time, how often do you engage in exercise? | - Often 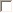 Sometimes 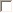 Never/Rarely |
| 22. In general, how would you rate your health today?  *(Please circle the number that best described your health today).* | 1. Very bad 2. Bad 3. Moderate 4. Good 5. Very Good |

**Work Related Questions II: Efforts and Rewards**

The following items refer to your present occupation. For each of the following statements, please indicate whether you strongly disagree, disagree, agree or strongly agree. We thank you for answering all statements.

|  | **Effort & reward scales** | | ***Strongly disagree*** | ***Disagree*** | ***Agree*** | ***Strongly agree*** |
| --- | --- | --- | --- | --- | --- | --- |
| **ERI 1** | I have constant time pressure due to a heavy work load. | | 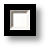 | 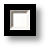 | 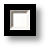 | 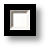 |
| **ERI 2** | I have many interruptions and disturbances while performing my job. | | 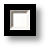 | 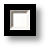 | 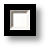 | 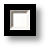 |
| **ERI 3** | I have a lot of responsibility in my job. | | 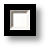 | 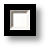 | 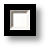 | 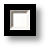 |
| **ERI 4** | I am often pressured to work overtime. | | 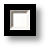 | 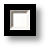 | 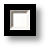 | 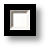 |
| **ERI 5** | My job is physically demanding. | | 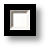 | 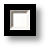 | 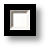 | 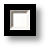 |
| **ERI 6** | | Over the past few years, my job has become more and more demanding. | 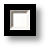 | 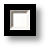 | 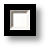 | 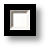 |
| **ERI 7** | | I receive the respect I deserve from my superior or a respective relevant person. | 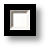 | 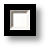 | 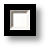 | 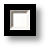 |
| **ERI 8** | I experience adequate support in difficult situations. | | 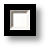 | 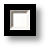 | 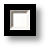 | 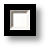 |
| **ERI 9** | I am treated unfairly at work. | | 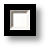 | 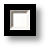 | 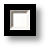 | 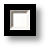 |
| **ERI 10** | My job promotion prospects are poor. | | 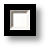 | 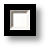 | 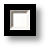 | 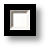 |
| **ERI 11** | I have experienced or I expect to experience an undesirable change in my work situation. | | 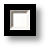 | 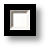 | 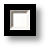 | 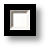 |
| **ERI 12** | My employment security is poor. | | 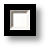 | 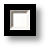 | 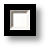 | 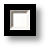 |
| **ERI 13** | | My current occupational position adequately reflects my education and training. | 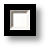 | 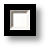 | 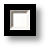 | 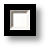 |
| **ERI 14** | | Considering all my efforts and achievements,  I receive the respect and prestige I deserve at work. | 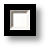 | 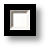 | 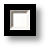 | 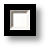 |
| **ERI 15** | | Considering all my efforts and achievements, my job promotion prospects are adequate. | 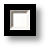 | 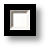 | 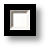 | 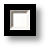 |
| **ERI 16** | | Considering all my efforts and achievements, my salary / income is adequate. |  |  |  |  |

**Work Related Questions III: Over-commitment**

The following items refer to your present occupation. For each of the following statements, please indicate whether you strongly disagree, disagree, agree or strongly agree. We thank you very much for answering all statement!!

|  | **Over-commitment scale** | ***Strongly disagree*** | ***Disagree*** | ***Agree*** | ***Strongly agree*** |
| --- | --- | --- | --- | --- | --- |
| **OC1** | I get easily overwhelmed by time pressures at work. |  |  |  |  |
| **OC2** | As soon as I get up in the morning I start thinking about work problems. |  |  |  |  |
| **OC3** | When I get home, I can easily relax and ‘switch off' work. |  |  |  |  |
| **OC4** | People close to me say I sacrifice too much for my job. |  |  |  |  |
| **OC5** | Work rarely lets me go, it is still on my mind when I go to bed. |  |  |  |  |
| **OC6** | If I postpone something that I was supposed to do today I'll have trouble sleeping at night. |  |  |  |  |
